# A blueprint of synergistic effect in Crataegus pinnatifida and obesity-related gut microbiota against obesity via systems biology concept

**DOI:** 10.1371/journal.pone.0332038

**Published:** 2025-10-01

**Authors:** Jinghui Xie, Haofang Guan, Maohui Liu, Weijun Ding

**Affiliations:** School of Basic Medical Sciences, Chengdu University of Traditional Chinese Medicine, Chengdu, Sichuan, PR China; University of Helsinki: Helsingin Yliopisto, FINLAND

## Abstract

**Background:**

Current obesity treatments include behavioral interventions, pharmacotherapy and surgery. Recently, the combination of ‘medicinal food’ products such as the plant Crataegus pinnatifida and its interaction with the gut microbiota has shown promise as an alternative therapeutic strategy to treat obesity.

**Methods:**

We obtained secondary metabolites (SMs) of obesity-related gut microbiota and Crataegus pinnatifida from gutMGene database and NAPSS database. bioinformatics analysis was used to elucidate key target and signaling pathways, whereas molecular docking (MD), molecular dynamics simulation and quantum chemical calculations identified crucial SMs involved in these pathways. The toxicity and physicochemical properties of these SMs were also assessed.

**Results:**

Phosphoinositide-3-kinase regulatory subunit 1 (PIK3R1), a key mediator in the phosphoinositide 3-kinase (PI3K)/ Protein Kinase B (Akt) pathway that is crucial for regulating insulin signaling and adipogenesis, emerged as the central hub within the PPI network. Strong binders to PIK3R1 were predicted to be quercetin, kaempferol and naringenin chalcone, suggesting their potential as therapeutic agents to treat obesity.

**Conclusion:**

The synergistic combination of Crataegus pinnatifida and the obesity-related gut microbiota holds promise as a novel therapeutic strategy for obesity by targeting PIK3R1 and modulating the PI3K/Akt signaling pathway. Further experimental validation is necessary to confirm these findings.

## 1. Introduction

Obesity is a complex metabolic disorder characterized by the dysregulation of energy homeostasis and excessive accumulation of body fat [1]. Obesity has emerged as a significant global public health challenge, contributing to approximately 2.8 million deaths annually [2]. This condition is a major risk factor for diseases such as cardiovascular disease, cognitive impairment and certain types of cancer [3–5]. Current treatment options are multifaceted, but pharmacological options are limited and often lack efficacy. Therefore, there is an urgent need to develop safer and more effective therapeutic strategies to control obesity.

Metabolic disorders including diabetes and obesity can potentially be treated with natural products via the modulation of blood glucose and lipid levels. These natural products also interact with the gut microbiota. The intestinal microbiota is a large and diverse group of microorganisms that reside in the human intestine, forming an ecosystem often referred to as a “metabolic organ” that communicates with the host. Beneficial bacteria in this system can produce and metabolize SMs, such as short-chain fatty acids, which are important for obesity regulation [6]. For example, probiotics and their metabolites can improve metabolic diseases by targeting the PI3K/AKT pathway [7].

Crataegus pinnatifida is a medicinal shrub that has the potential to treat a range of health conditions, as it has been reported to have antiviral and anti-inflammatory properties and is able to prevent cardiovascular diseases [8–10]. This plant can be used either as food or as a medicine and has been extensively utilized in China in food preparations aimed at weight loss. Crataegus pinnatifida has also shown promise in the modulation of the gut microbiota. For example, Crataegus pinnatifida polysaccharides alleviate colitis in mice by regulating their gut flora and producing beneficial metabolites [11]. Additionally, its polysaccharides and proanthocyanidins improve lipid metabolism disorders and nonalcoholic fatty liver disease by modulating beneficial gut microorganisms [12,13].

The gut microbiota, the largest and most diverse group of microorganisms residing in the human intestine, forms a complex ecosystem often referred to as a “metabolic organ” [14]. This ecosystem communicates with the host and derives energy by metabolizing various host-derived substances. Notably, beneficial bacteria within this system can produce and metabolize SMs such as vitamins and short-chain fatty acids, which play vital roles in obesity regulation [15]. Recent studies have indicated that certain probiotics and their metabolites, such as short-chain fatty acids, can improve metabolic diseases such as diabetes by targeting the PI3K/AKT pathway [16,17]. Thus, our study investigated the combined regulatory effects of Crataegus pinnatifida-derived SMs and obesity-related gut microbiota on obesity. Using network pharmacology and bioinformatics analysis, we identified key gene targets, signaling pathways, SMs, and microbial species involved in obesity regulation. The advent of high-throughput sequencing technologies has revolutionized the life sciences, providing precise insights into disease mechanisms and target genes, thereby facilitating the development of targeted therapies. Network pharmacology, an emerging discipline that integrates systems biology and genomics, has demonstrated its potential in linking diseases, drugs, and targets, thus advancing clinical drug development. Previous studies have successfully utilized network pharmacology combined with gut microbiota analysis to elucidate the role of the intestinal flora in hyperlipidemic regulation [18]. Similarly, Tai et al. leveraged these approaches to predict obesity-related targets and identify Chinese herbal medicines with therapeutic potential against obesity [19].

**Fig 1:**
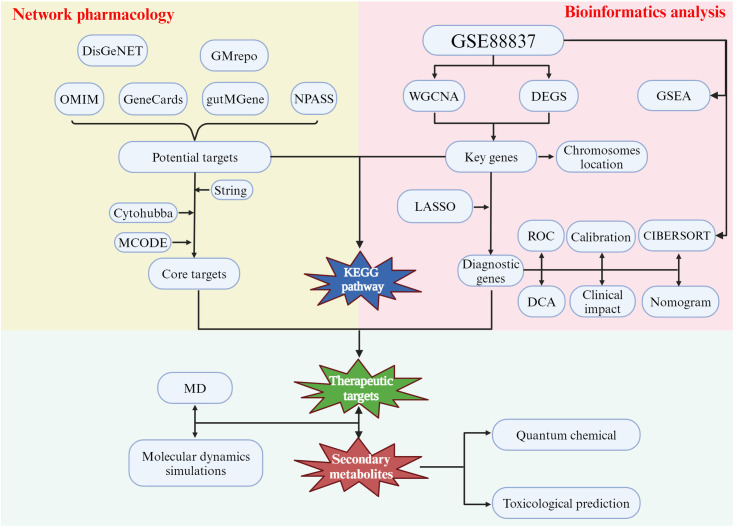
The flowchart of this study.

Hence, we aimed to identify key SMs that can mitigate obesity by exploring those associated with Crataegus pinnatifida and the obesity-related gut microbiota. We also used network pharmacology and bioinformatics to identify key gene targets and signaling pathways for these SMs with the final objective of determining the feasibility of using Crataegus pinnatifida and obesity-related gut microbiota in the management of obesity. We summarize the analysis process of the entire study in Fig 1.

## 2. Methods

### 2.1 Identification of SMs and targets of obesity-related gut microbiota

Obesity-related gut microbiota were identified by querying data from healthy and obese individuals in the GMrepo database (screening threshold |LDA score| > 2) [20]. SMs of these microbes were obtained from the gutMGene v1.0 database (https://bio-annotation.cn/gutmgene/) [21]. Standard simplified molecular input line entry system representations of these SMs were retrieved from the PubChem database (https://pubchem.ncbi.nlm.nih.gov/), and potential targets were predicted via Swiss Target Prediction (https://www.swisstargetprediction.ch/).

### 2.2 Identification of SMs and targets of Crataegus pinnatifida

The SMs of Crataegus pinnatifida were obtained from the Natural Product Activity & Species Source database (http://bidd.group/NPASS/). All SMs were accepted by Lipinski’s rule [22]. The SMILES for these SMs were retrieved from PubChem, and their potential biological targets were identified via SwissTargetPrediction.

### 2.3 Identification of combined targets for Crataegus pinnatifida and obesity-related gut microbiota for obesity treatment

Obesity-related targets were obtained from the combination of targets retrieved via the keywords “obesity” from DisGeNET (https://www.disgenet.org/), GeneCards (https://www.genecards.org/) and OMIM (https://www.omim.org/). To identify which of these targets were common to Crataegus pinnatifida and obesity-related gut microbiota, we used the *VennDiagram* (version 1.7.3) R package [23].

### 2.4 Construction of a protein-protein interaction network

The STRING database (https://string-db.org/) was used to construct a PPI network (confidence threshold of 0.9). Strong interactions within the network were visualized via Cytoscape 3.10.3. Two methods in Cytoscape were used to identify core targets. One was the CytoHubba plugin, which includes 12 algorithms with MCC, Degree and Closeness algorithms used to prioritize core genes. The other was the MCODE algorithm, where the subnetwork with the highest score was selected. We used the intersection of these methods to determine the core targets for the treatment of obesity using Crataegus pinnatifida and obesity-related gut microbiota.

### 2.5 Gene Ontology (GO) and Kyoto Encyclopedia of Genes and Genomes (KEGG) enrichment analysis

We performed GO (www.geneontology.org/) and KEGG (www.genome.jp/kegg/pathway.html) analysis via the *clusterProfiler*(version 4.10.0) package [24]. Visualization of the enriched KEGG pathways was conducted with the *pathview* (version 1.42.0) R package [25]. A p value < 0.05 was considered statistically significant.

### 2.6 Downloading and processing the obesity dataset

The obesity dataset GSE88837 was retrieved from the GEO database via the *GEOquery* (version 2.70.0) R package [26,27]. This dataset comprises samples from *Homo sapiens*, specifically from visceral adipose tissue, and includes 30 adolescents (15 lean and 15 obese). We standardized the dataset GSE88837 and normalized the annotation probes using the *limma* (version 3.58.1) R package [28]. Differentially expressed genes (DEGs) were identified on the basis of the following criteria: p value < 0.05 and |log fold change (FC)| > 0.585.

### 2.7 Weighted gene co-expression network analysis (WGCNA)

The top 5000 genes were filtered and selected for WGCNA analysis based on the p-value of differential analysis. WGCNA was performed via the *WGCNA* (version 1.72−1) R package [29]. Initially, we calculated the correlation coefficients between all pairs of genes and applied weighted values to ensure that the network followed a scale-free topology. We then constructed a hierarchical clustering tree on the basis of gene correlations, where different branches represent distinct gene modules (depicted in different colors). According to the principle of a scale-free network, soft thresholds (power = 24, R^2^ = 0.87) were selected to construct a scale-free co-expression network. Module significance was calculated by setting the minimum number of module genes to 50, and the module merging cut height to 0.25. Finally, we identified the module with the highest correlation, and its genes were intersected with the DEGs to pinpoint key genes.

### 2.8 Gene Set Enrichment Analysis (GSEA)

The gene sets used in GSEA were obtained from the C2 collections in the MSigDB database using the msigdbr function in the *msigdbr* (version 7.5.1) R package. Gene sets with NES > 0 and padj < 0.05 were considered significantlyenriched. GSEA evaluates the distribution trend of genes within predefined gene sets across a ranked gene list on the basis of their phenotypic relevance, determining their contribution to the observed phenotype.

### 2.9 Screening of core diagnostic genes

The candidate genes were those at the intersection of the DEGs and module genes identified via WGCNA. We performed the least absolute shrinkage and selection operator (LASSO) via the *glmnet* (version 4.1–8) R package [30].

### 2.10 Construction of Receiver Operating Characteristic (ROC) curves and nomograms for diagnostic genes

The diagnostic performance of the core diagnostic genes was evaluated by constructing ROC curves using the *pROC* (version 1.18.5) package [31]. We developed a nomogram using the *rms* (version 6.7−1) R package to visualize and predict diagnostic accuracy [32]. The accuracy of the nomogram was assessed via calibration curves, and its clinical utility was evaluated via decision curve analysis.

### 2.11 Immune infiltration analysis

The *GSVA* (version 1.50.0) and *GSEABase* (version 1.64.0) packages were utilized to perform single-sample gene set enrichment analysis (ssGSEA) to obtain immune cells. Differences in these scores between the diseased group and the control group were compared via the Wilcoxon rank-sum test. Spearman correlation analysis was employed to assess the relationships between hub genes and immune cells or immune function. A p value of less than 0.05 was considered statistically significant.

### 2.12 Molecular docking

The crystal structures of the target proteins were obtained from the Protein Data Bank (https://www.rcsb.org/). Using PyMOL-2.1.0 software, we optimized them by removing water molecules and small molecule ligands, followed by hydrogenation and charge adjustments with AutoDock Tools-1.5.6, saving the results in pdbqt format. The drug structures were sourced from the PubChem database. We used Vina-2.0 in PyRx software to calculate the binding energies and generate output files. The binding results were visualized with PyMOL software.

### 2.13 Quantum chemical (QC) calculations

The Gaussian16 package was used to perform QC calculations on the SMs, employing the B3LYP functional with D3 empirical dispersion correction. We optimized the geometries and conducted frequency calculations using the def2-SVP basis set. Multiwfn 3.8 software was then used to extract cube files and orbital energies for the highest occupied molecular orbital (HOMO) and lowest unoccupied molecular orbital (LUMO). Finally, ChimeraX 1.7 was used to visualize the orbital diagrams.

### 2.14 MD simulations

To predict the binding of SMs to the core targets, MD simulations were conducted via the Desmond program. The OPLS2005 force field was employed to parameterize the interactions, whereas the TIP3P model simulated water molecules. The SM-target complexes were immersed in a cubic water box. To maintain system neutrality, 0.15 M chloride and sodium ions were added. Prior to the simulation, the energy was minimized over 50,000 steps via the steepest descent method to achieve an initial stable state. Following energy minimization, two equilibrium stages were performed: 50,000 steps of NVT equilibrium were followed by an equal number of NPT equilibrium steps. During these stages, the heavy atom positions were restricted to facilitate system adaptation to the simulation environment. An unconstrained 100 ns simulation was subsequently conducted to observe the dynamic behavior of the complexes in the free state. Trajectory data, including energy and coordinates, were saved every 10 ps. Throughout the simulations, the temperature and pressure were maintained at 300 K and 1 bar, respectively, to reproduce physiological conditions.

### 2.15 Pharmacokinetics and toxicology prediction

Pharmacokinetic profiles of selected SMs were predicted SwissADME website (http://www.swissadme.ch/). The toxicity of the selected SMs was assessed via ProTox 3.0 (https://tox.charite.de/protox3/), and the toxicity level, lethal dose, hepatotoxicity, immunotoxicity, mutagenicity, carcinogenicity and cytotoxicity were evaluated.

### 2.16 Statistical analysis

All data calculations and statistical analyzes were performed via R programming (https://www.r-project.org/, version 4.3.2). For the comparison of two groups of continuous variables, the statistical significance of normally distributed variables was estimated by independent Student’s t test, and the Wilcoxon rank sum test was used to analyze differences between non-normally distributed variables. p < 0.05 was considered statistically significant. The R code for this study is in S1 File.

## 3. Results

### 3.1 Network pharmacology analysis

#### 3.1.1 Crataegus pinnatifida SMs and their targets.

From the NPASS database, we identified 55 Crataegus pinnatifida-derived SMs, all of which complied with Lipinski’s rule of five. Using SwissTargetPrediction, we discovered 544 targets associated with these SMs, which are therefore considered potential regulatory targets.

#### 3.1.2 Obesity-related gut microbiota SMs and their targets.

We identified 126 obesity-related gut microbiota from the GMrepo database. These microbes were cross-referenced with the gutMgene database, leading to the identification of 71 SMs. The targets of these 610 SMs were determined via SwissTargetPrediction.

#### 3.1.3 Crataegus pinnatifida and obesity-related gut microbiota combined targets.

**Fig 2:**
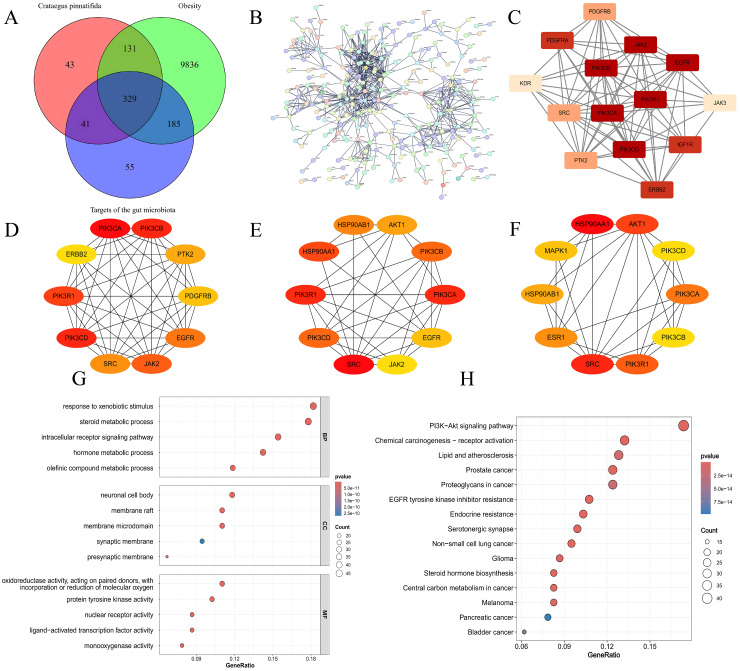
Network pharmacology analysis. **(A)**. 329 potential therapeutic targets for treating obesity according to Venny. **(B)**. PPI network of the therapeutic targets generated via STRING. **(C)**. The highest scoring subnetwork in MCODE. **(D-F)**. MCC, Dgree, Closeness algorithms for confirming core targets. **(G)**. Enrichment of biological process, cell composition, and molecular function terms. **(H)**. KEGG pathway enrichment analysis of the top 15.

We compiled 10,481 obesity-related targets from the DisGeNET, GeneCards and OMIM databases. The intersection of these targets with SMs derived from Crataegus pinnatifida, obesity-related gut microbiota metabolites and obesity-related targets yielded 329 targets (Fig 2A).

#### 3.1.4 Construction of the PPI network and identification of core targets.

These 329 targets were input into the STRING database (confidence threshold of 0.9), resulting in 254 targets for further analysis. These targets were then used to construct a PPI network (Fig 2B). The MCODE plugin was used for topological analysis, selecting the highest-scoring subnet for further core gene analysis (Fig 2C). The network data were imported into Cytoscape software, and core targets were identified via the MCC, degree and closeness algorithms within the cytoHubba plugin (Fig 2D-F). The intersection of the results from these two plugins revealed that PIK3R1, PIK3CB, SRC, PIK3CA and PIK3 CD are core targets for the combined treatment of obesity via Crataegus pinnatifida andgut microbiota.

#### 3.1.5 GO and KEGG enrichment analysis.

GO and KEGG enrichment analysis were performed on these 254 targets. GO analysis indicated that these targets are involved primarily in biological processes such as response to xenobiotic stimulus, steroid metabolic process, and hormone metabolic process; cellular components such as neuronal cell body, membrane raft and membrane microdomain; and molecular functions such as protein tyrosine kinase activity, nuclear receptor activity and ligand−activated transcription factor activity (Fig 2G). KEGG pathway analysis revealed the significant involvement of these targets in PI3K−Akt signaling pathway, the Endocrine resistance and Steroid hormone biosynthesis (Fig 2H). The completem results of GO and KEGG analysis can be found in S1 Table.

### 3.2 Bioinformatics validation

#### 3.2.1 Determination of DEGs in obesity.

**Fig 3:**
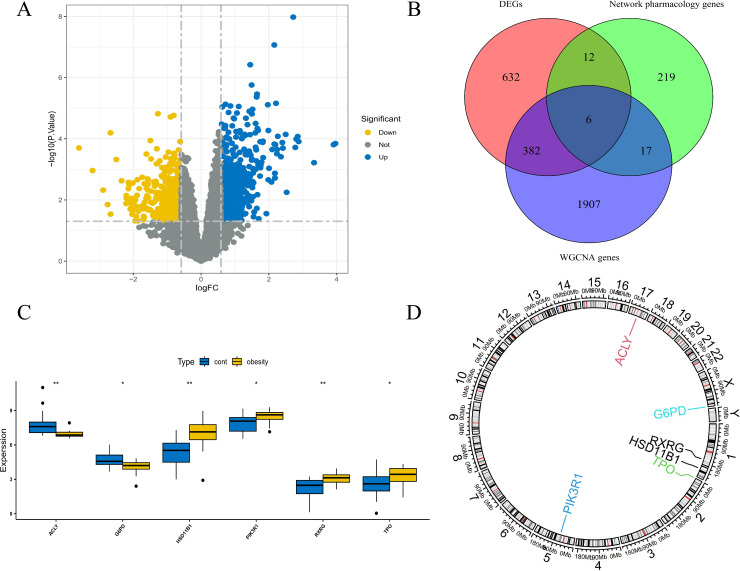
Identification of key genes and correlation analysis. **(A)**. Volcano plot of DEGs. The blue dots represent DEGs that are highly expressed in the obese group, and the yellow dots represent DEGs that are expressed at low levels in the obese group. **(B)**. Identification of key genes via a Venn diagram. **(C)**. Box plot of 6 key genes. **(D)**. Chromosome localization map of key genes.

The thresholds for DEGs were set at |log2(FC)| > 0.585 and p value < 0.05 and were visualized via volcano plots (Fig 3A). We identified 1,036 DEGs (575 upregulated and 461 downregulated). The differentially expressed genes can be found in S2 Table.

#### 3.2.2 GO and KEGG enrichment analysis of key genes.

**Fig 4:**
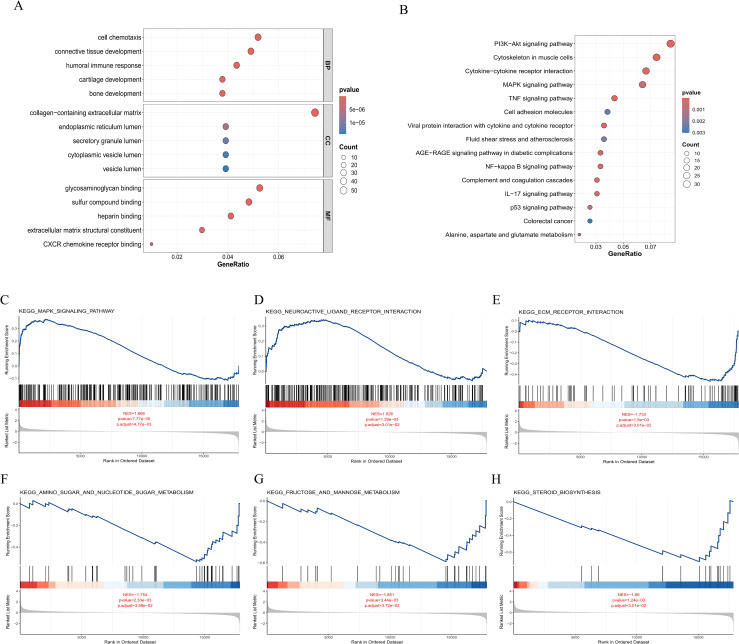
Enrichment analysis. **(A)**. GO enrichment of biological processes, cell composition, and molecular function. **(B)** KEGG pathway enrichment analysis of the top 1036 genes. **(C-H)** GSEA revealed that obesity significantly affects the signaling pathways of the MAPK signaling pathway **(C)**, neuroactive ligand–receptor interaction **(D)**, ECM receptor interaction **(E)**, amino sugar and nucleotide sugar metabolism **(F)**, fructose and mannose metabolism **(G)**, and steroid biosynthesis **(H)**. The GSEA screening criterion was padj < 0.05.

We conducted GO and KEGG analysis on 1036 differential genes from the dataset. GO analysis revealed that these genes are involved primarily in biological processes such as cell chemotaxis, connective tissue development and humoral immune response; cellular components such as collagen−containing extracellular matrix, endoplasmic reticulum lumen and secretory granule lumen; and molecular functions such as glycosaminoglycan binding, heparin binding and extracellular matrix structural constituent (Fig 4A). KEGG pathway analysis indicated that these key genes play significant roles in the PI3K−Akt signaling pathway, Cytokine−cytokine receptor interaction and MAPK signaling pathway (Fig 4B). The completem results of GO and KEGG analysis can be found in S3 Table.

#### 3.2.3 GSEA enrichment analysis.

To evaluate the impact of gene expression levels on obesity, we utilized GSEA to explore the relationships between gene expression and related biological processes and pathways. The results showed that all genes in the dataset were significantly enriched in signaling pathways such as KEGG_MAPK_SIGNALING_PATHWAY (Fig 4C), KEGG_NEUROACTIVE_LIGAND_RECEPTOR_INTERACTION (Fig 4D), KEGG_ECM_RECEPTOR_INTERACTION. (Fig 4E), KEGG_AMINO_SUGAR_AND_NUCLEOTIDE_SUGAR_METABOLISM (Fig 4F), KEGG_FRUCTOSE_AND_MANNOSE_METABOLISM (Fig 4G), and KEGG_STEROID_BIOSYNTHESIS (Fig 4H).

#### 3.2.4 WGCNA results.

**Fig 5:**
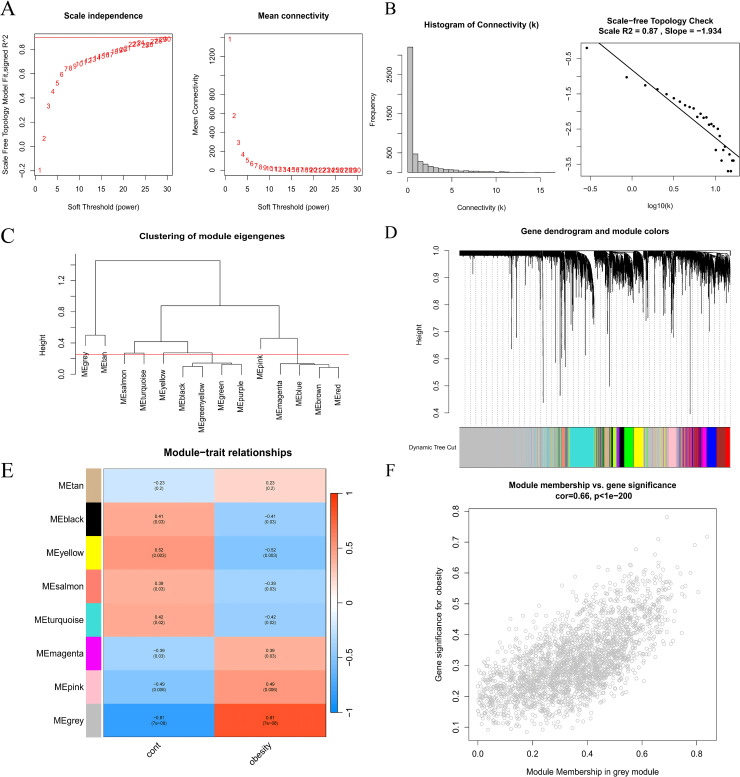
WGCNA of GSE88837. **(A)**. Scale-free network display of the optimal soft threshold in WGCNA. The left image is the optimal soft threshold, and the right image is the network connectivity under different soft thresholds. **(B)**. Histogram of connectivity distribution. The scale-free topology is checked at a soft threshold of 24. **(C)**. Display of the module aggregation results. **(D)**. Clustering results. The upper part is a hierarchical clustering dendrogram, and the lower part is a gene module. **(E)**. Correlation analysis results between the variance gene clustering module and the control group and obesity group. **(F)**. Scatter plot between gene salience (GS) and module members (MM) in grey.

For compatibility with the scale-free network, a soft threshold of = 24 (scale-free R^2^ = 0.87; slope = −1.934) was used (Fig 5A–5B). Construct a co-expression network based on the optimal soft threshold, cluster the genes through the clustering tree and annotate the grouping information (Fig 5C). The co-expression matrix was built in one step, and eight gene modules were obtained using dynamic hybrid shearing (Fig 5D). These genes clustered into eight modules: MEtan, MEblack, MEyellow, MEsalmon, MEturquoise, MEmagenta, MEpink and MEgray. From the module with the highest correlation (grey module), we selected 2,312 genes for further analysis. The grey module shows the strongest association (cor) with obesity (cor = 0.81; p = 7e-08) (Fig 5E). The scatter plot shows a strong correlation between GS and MM in the “grey” module (cor = 0.66,p < 1e-200) (Fig 5F). The intersection of these 2,312 module genes and 1,036 DEGs yielded 388 significant genes, whereas the intersection between these 388 genes and 254 network pharmacology targets identified 6 genes for further analysis (HSD11B1, RXRG, ACLY, G6PD, TPO and PIK3R1) (Fig 3B). We also constructed a box plot to compare the differences in the expression of key genes between the control group and the obese group (Fig 3C). The chromosomal locations of these genes were mapped via the *circlize* (version 0.4.15) package, which revealed a predominant distribution on chromosomes 1 (Fig 3D).

#### 3.2.5 Core diagnostic genes obtained through a machine learning algorithm.

**Fig 6:**
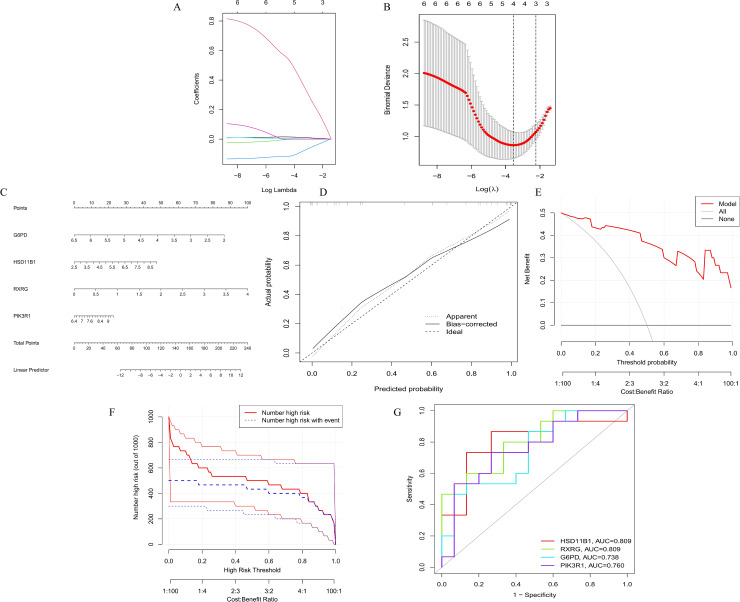
Machine learning algorithm and nomogram model construction for obesity. **(A-B)**. Variable trajectory diagram (A) and diagnostic model diagram (B) of the LASSO regression. **(C)** Nomogram to predict obesity risk. **(D)** Calibration curve of the obesity diagnostic model. DCA (E) and clinical impact curve (F) curves were used to assess the practical efficacy of the nomogram. **(G)** ROC curve based on the expression of core diagnostic genes.

To identify the core diagnostic genes among these genes, we applied a machine learning algorithm. The LASSO regression models were built from data from obese and normal tissues, and the λ analysis indicated that the model achieved optimal predictive accuracy for obesity at λ = 4, leading to the identification of 4 core diagnostic genes, including HSD11B1, RXRG, G6PD and PIK3R1 (Fig 6A-B).

#### 3.2.6 ROC curves and nomogram results of the core diagnostic genes.

To further assess the diagnostic utility of these genes, we constructed a nomogram model (Fig 6C). This model, along with its calibration curves (Fig 6D), decision curve analysis (DCA) (Fig 6E), and clinical impact curve (Fig 6F), confirmed that it has a robust ability to predict obesity. We also generated receiver operating characteristic (ROC) curves for the 4 core diagnostic genes, which all exhibited strong diagnostic performance, with area under the curve (AUC) values exceeding 0.7 (Fig 6G), where HSD11B1 and RXRG had the highest diagnostic value (AUC value = 0.809).

#### 3.2.7 Immune infiltration analysis.

**Fig 7:**
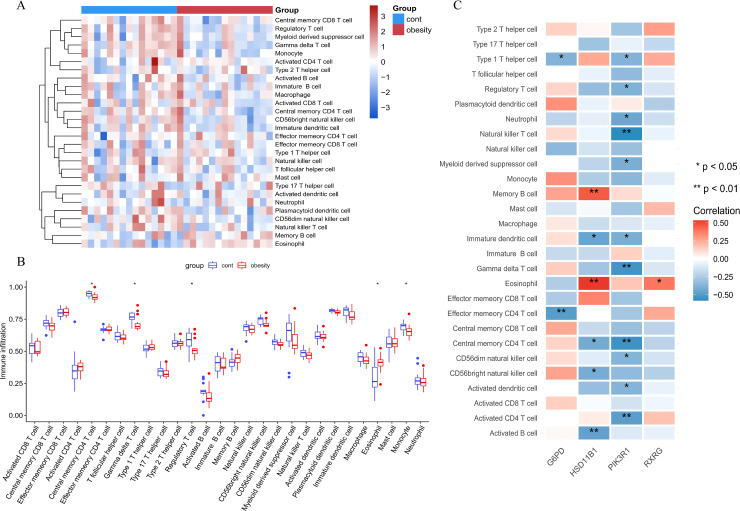
Immune infiltration analysis. **(A)** Heatmap showing the immune scores of 28 immune cells. **(B)** Comparison of immune cell infiltration levels between the Con group and the Obese group. **C.** Correlations between immune cell infiltration and the three hub genes. * p < 0.05, ** p < 0.01.

Using the ssGSEA algorithm, we analyzed the correlations between 28 types of immune cells and the normal and obese groups (Fig 7A-7B). The immune infiltration analysis results were presented in a heatmap and group comparison boxplots, which highlight the statistically significant differences in the infiltration abundance of central memory CD4 T cells, gamma delta T cells, regulatory T cells, eosinophils and monocytes (P < 0.05). The relationship between core diagnostic genes and the abundance of immune cell were showed in correlation plots (Fig 7C).

### 3.3 Network pharmacology and bioinformatics joint analysis

#### 3.3.1 Identification of obesity treatment targets by Crataegus pinnatifida and the gut microbiota.

**Fig 8:**
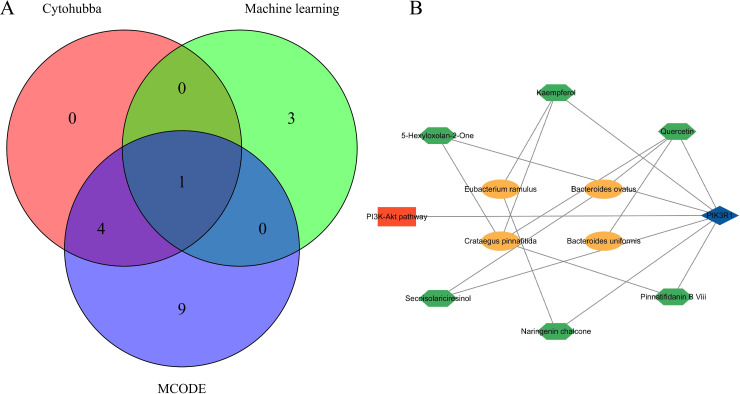
Joint analysis. **(A)**. Venn diagram identifying PIK3R1 as a core target for obesity treatment. **(B)** PPI diagram of the target-pathway-SM-gut microbiota/Crataegus pinnatifida. The blue rhombus represents targets, the Orange rectangle represents pathways, the yellow ovals represent the gut microbiota and Crataegus pinnatifida, and the green hexagons represent SMs.

Through network pharmacology analysis, we revealed that SMs of Crataegus pinnatifida and obesity-related gut microbiota could target obesity by interacting with PIK3R1, PIK3CB, SRC, PIK3CA and PIK3 CD. Complementary bioinformatics analysis revealed four key targets associated with obesity onset: HSD11B1, RXRG, G6PD and PIK3R1. By combining the targets identified via both approaches, PIK3R1 was selected as the likely protein target by which Crataegus pinnatifida and obesity-related gut microbiota may be able to treat obesity (Fig 8A). We constructed a PPI network to describe the network of target-pathway-SM-gut microbiota/Crataegus pinnatifida (Fig 8B).

#### 3.3.2 Molecular docking results.

**Fig 9:**
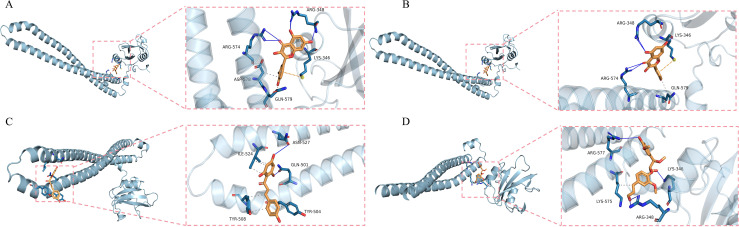
Molecular docking results. The four SMs that docked well with PIK3R1 were quercetin **(A)**, kaempferol **(B)**, naringenin chalcone **(C)**, pinnatiflidin B Viii **(D)**.

To evaluate the potential of the identified SMs in targeting PIK3R1, we conducted molecular docking studies. Among the six SMs predicted to interact with PIK3R1, four were predicted to have strong binding affinities (binding energies of less than −5 kcal/mol) (Fig 9A-9D, S4 Table). Notably, quercetin and kaempferol were predicted to have a binding energy of −6.9 kcal/mol, whereas naringenin chalcone had a binding energy of −6.3 kcal/mol. Therefore, these three metabolites were selected for further analysis.

#### 3.3.3 Quantum chemical calculations.

**Fig 10:**
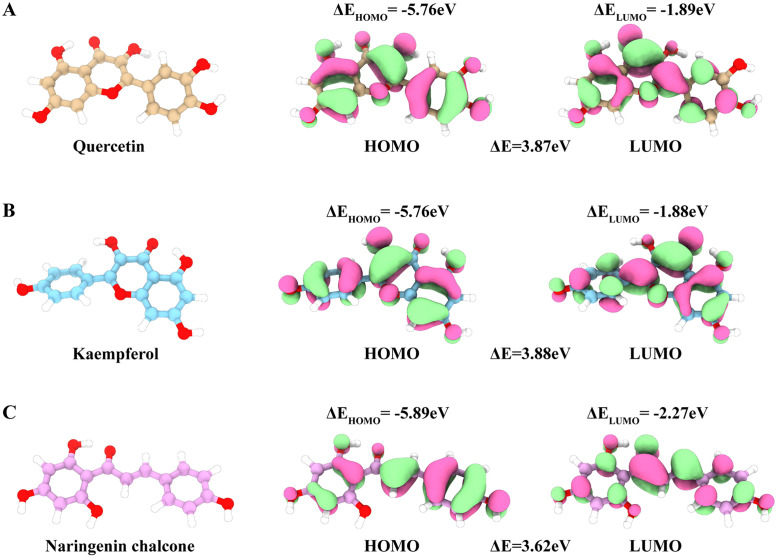
Molecular structure and HOMO–LUMO orbital diagram of quercetin (A), kaempferol (B) and naringenin chalcone (C).

The HOMO and LUMO energy levels provide insight into the electron donation and acceptance capabilities of a molecule. Gaussian calculations revealed the HOMO‒LUMO energies of three SMs, quercetin, kaempferol and naringenin chalcone (Fig 10A-10C). These calculations suggest that naringenin chalcone has the smallest predicted energy gap and may be more effective as an electron donor, possibly being more reactive than quercetin and kaempferol are.

#### 3.3.4 Molecular dynamics simulation.

**Fig 11:**
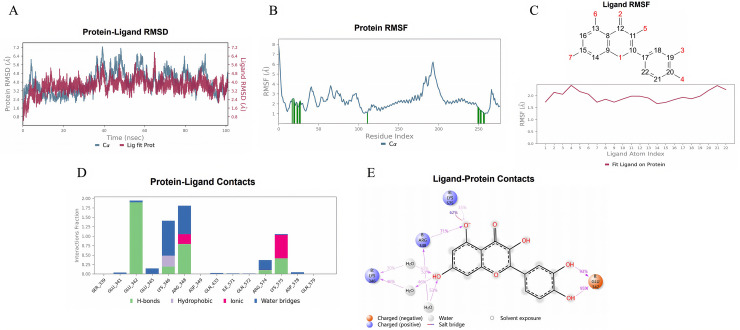
Molecular dynamics simulation results of quercetin with PIK3R1. (A) RMSD. (B) Protein RMSF. (C) Ligand RMSF. (D, E) The connection between the ligand and protein.

**Fig 12:**
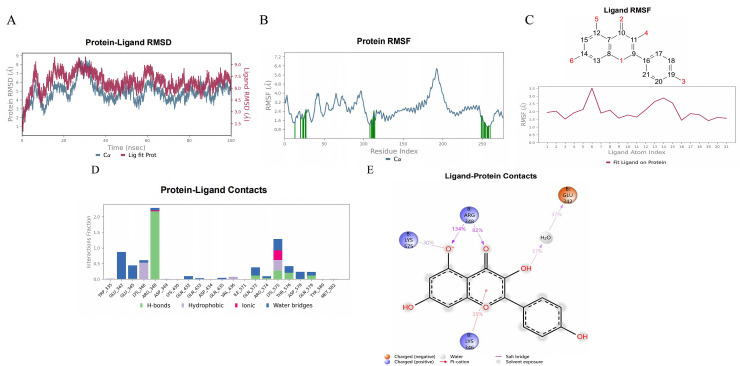
Molecular dynamics simulation results of kaempferol with PIK3R1. (A) RMSD. (B) Protein RMSF. (C) Ligand RMSF. (D, E) The connection between the ligand and protein.

We conducted a 100 ns molecular dynamics simulation to study the interactions between the SMs and the PIK3R1 protein. The root mean square deviation (RMSD) of the protein-SM complexes indicated that the system reached a stable state at approximately 40 ns (Fig 11A, 12A, 13A). Consequently, trajectories from 40 to 100 ns were selected for further analysis. We extracted root mean square fluctuation (RMSF) data and calculated the corresponding B-factors for both proteins and small molecules. The RMSF and B factor plots demonstrated that the overall protein structure maintained low flexibility, with RMSF values predominantly below 3.0 Å, suggesting high structural stability throughout the simulation (Fig 11B, 12B, 13B). Focusing on the stable interval (40–100 ns), we analyzed the interaction patterns between the PIK3R1 protein and the SMs.

**Fig 13:**
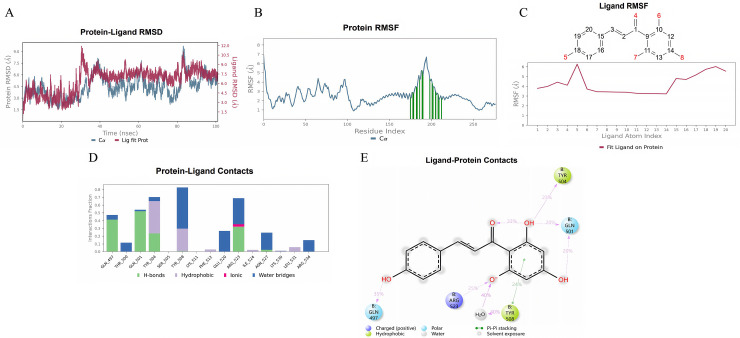
Molecular dynamics simulation results of naringenin chalcone with PIK3R1. **(A)** RMSD. **(B)** Protein RMSF. **(C)** Ligand RMSF. **(D, E)** The connection between the ligand and protein.

In the RMSF diagram of small molecules (Fig 11C, 12C, 13C), most atoms were in a relatively stable state during the simulation. The key residues involved in binding with quercetin and kaempferol include Glu 342, Lys 346, Arg 348 and Lys 575 (Fig 11D, 12D). These interactions, which involve water bridges, hydrogen bonds, salt bridges and hydrophobic contacts, significantly increase the stability and affinity of the complexes. The binding of quercetin to PIK3R1 occurs mainly through the phenolic hydroxyl group and the surrounding Glu 342, Lys 346, Arg 348 and Lys 576 residues of the protein to form a stable hydrogen bond interaction network (Fig 11E). The phenolic hydroxyl groups of kaempferol also established stable hydrogen bonds with Glu 342, Arg 348, and Lys 575, and its aromatic skeleton engaged in Pi-cation interactions with the Lys 346 side chain, further stabilizing the complex (Fig 12E). Naringenin chalcone interacted with residues Gln 497, Gln 501, Tyr 504, Tyr 508 and Arg 523 through water bridges, hydrogen bonds, and hydrophobic interactions (Fig 13D). Its phenolic hydroxyl groups formed stable hydrogen bonds with Gln 497, Gln 501, Tyr 504 and Arg 523, whereas its aromatic ring established Pi‒Pi interactions with Tyr 508, stabilizing small molecule‒protein binding (Fig 13E).

#### 3.3.5 The pharmacokinetic properties and toxicity of key SMs.

SwissADME results showed that these three SMs all had high gastrointestinal absorption rates and no blood-brain barrier permeability. However, they all inhibited isoforms CYP1A2 and CYP3A4 ability. This suggests that there may be a risk of drug interaction.The toxicological analysis results suggested that kaempferol has no activity against any of the tested toxins. In contrast, quercetin exhibited active toxicity in the carcinogenicity and mutagenicity assays, remaining inactive for other toxicities. Naringenin chalcone had active toxicity in the immunotoxicity but was inactive for the other assessed toxicities (Table 1).

**Table 1:** The pharmacokinetic properties and toxicity of key SMs.

	Kaempferol	Quercetin	Naringenin chalcone
GI absorption	High	High	High
BBB permeant	No	No	No
Pgp substrate	No	No	No
Lipinski	Yes	Yes	Yes
CYP1A2 inhibitor	Yes	Yes	Yes
CYP2C19 inhibitor	No	No	No
CYP2C9 inhibitor	No	No	Yes
CYP2D6 inhibitor	Yes	Yes	No
CYP3A4 inhibitor	Yes	Yes	Yes
Toxicity Class	5	3	5
LD50 (mg/kg)	3919	159	3600
Hepatotoxicity (Probability)	Inactive (0.68)	Inactive (0.69)	Inactive (0.60)
Carcinogenicity (Probability)	Inactive (0.72)	Active (0.68)	Inactive (0.67)
Immunotoxicity (Probability)	Inactive (0.96)	Inactive (0.87)	Active (0.82)
Mutagenicity (Probability)	Inactive (0.52)	Active (0.51)	Inactive (0.80)
Cytotoxicity (Probability)	Inactive (0.98)	Inactive (0.99)	Inactive (0.74)

## 4. Discussion

Obesity is a metabolic disorder, and effective strategies for its prevention and treatment are needed. The metabolites produced by the obesity-related gut microbiota and those derived from Crataegus pinnatifida can be used to manage obesity. Through network pharmacology analysis and bioinformatics analysis, we identified key pathways and targets for Crataegus pinnatifida and obesity-related gut microbiota for obesity treatment.

In the present study, network pharmacology results showed that Crataegus pinnatifida combined with the obesity-related gut microbiota may treat obesity by targeting PIK3R1, PIK3CB, SRC, PIK3CA, PIK3 CD. Through the bioinformatics analysis, we identified HSD11B1, RXRG, G6PD, and PIK3R1 as core genes for the interplay between Crataegus pinnatifida and the obesity-related gut microbiota in obesity regulation. By integrating network pharmacology and bioinformatics analysis, we identified PIK3R1 as the ultimate target for Crataegus pinnatifida combined with the obesity-related gut microbiota in the treatment of obesity. Consistent with our bioinformatics findings, previous study has demonstrated elevated PIK3R1 expression in white adipose tissue of high-fat diet-induced obese mice [33]. Notably, this study revealed that reduced PIK3R1 expression can ameliorate insulin resistance and adipose tissue macrophage accumulation in obese mice.

GO and KEGG results showed that Crataegus pinnatifida and obesity-related gut microbiota potential therapeutic targets were mainly involved in PI3K−Akt signaling pathway, the Endocrine resistance and Steroid hormone biosynthesis in network pharmacology analysis. In addition, we performed KEGG enrichment analysis on the differentially expressed genes in obese patients in the dataset and found that these obesity-related differentially expressed genes were mainly involved in PI3K−Akt signaling pathway, Cytokine−cytokine receptor interaction and MAPK signaling pathway. These results indicate that PIK3R1 and PI3K−Akt signaling pathways are the core targets and pathways for Crataegus pinnatifida and obesity-related gut microbiota in the treatment of obesity. This pathway is a key component of insulin signaling, where the effects of insulin on adipogenesis are mediated by PI3K/Akt signaling [34]. In mature adipocytes, activation of the PI3K-AKT pathway enhances glucose uptake and lipid biosynthesis, reinforcing the importance of this pathway in metabolic regulation [35,36]. PIK3R1, which encodes the PI3K regulatory subunit p85α, is an important component of the PI3K/AKT signaling pathway. Studies have shown that PIK3R1 can positively regulate the activation of PI3K/AKT [37]. Another study confirmed that PPARγ can directly promote the expression of PIK3R1 in adipocytes, leading to insulin-induced AKT activation, thereby promoting adipogenesis in adipocytes [38]. In addition, in vitro experiments confirmed that overexpression of PIK3R1 can activate the PI3K/Akt signaling pathway, thereby relieving the inhibition of human bone marrow mesenchymal stem cell adipogenesis mediated by miR-100-3p [39]. This suggests that PIK3R1 can promote human bone marrow mesenchymal stem cell adipogenesis by activating the PI3K/Akt signaling pathway.

Finally, molecular docking predicted that, among the eight predicted SMs, quercetin, kaempferol and naringenin chalcone would have the highest binding affinity to PIK3R1, which is supported by MD simulations. Quercetin is one of the most abundant flavonoids and is widely found in vegetables and fruits [40]. A large number of in vitro and in vivo studies have confirmed that quercetin can inhibit the occurrence of obesity. Studies have shown that in C57BL/6J mice, a high-fat diet supplemented with different concentrations of quercetin can reduce body weight, liver fat accumulation, and liver triglyceride levels [41]. Kaempferol is a natural flavonoid with beneficial biological properties such as anti-inflammatory and antioxidant properties [42]. An in vitro study showed that kaempferol inhibited the adipogenic differentiation of 3T3-L1 cells by inhibiting the expression of Cebpa, a gene regulating adipocyte differentiation, and reduced lipid accumulation in mature adipocytes by promoting the transcription of lipid metabolism-related genes Pnpla2 and Lipe, thereby achieving the purpose of treating obesity [43]. Naringenin chalcone is a flavonoid extracted from tomato peel that has anti-allergic, antioxidant and anti-inflammatory effects. It can improve the metabolic function of adipocytes and exert an insulin sensitizing effect by promoting the expression of adiponectin gene and genes related to mitochondrial energy metabolism [44].

In our study, quercetin was derived mainly from Bacteroides uniformis, Bacteroides ovatus, and C. pinnafitida. Analysis of the GMrepo database revealed that Bacteroides uniformis and Bacteroides ovatus exhibited LDA scores of −4.198 and −4.078, respectively, demonstrating significant enrichment in healthy populations. As prominent members of the Bacteroidetes phylum, these anaerobic bacteria colonize the mammalian gastrointestinal tract where they establish stable, symbiotic relationships with their host [45]. Notably, they represent the most abundant Gram-negative bacterial species in the human gut microbiota. Emerging evidence positions Bacteroides uniformis and Bacteroides ovatus as promising next-generation probiotic candidates [46,47]. Experimental studies have demonstrated that Bacteroides uniformis CECT 7771 can improve the metabolic and immune dysfunction induced by high-fat diet in obese mice due to intestinal flora imbalance [48]. Another study showed that Bacteroides ovatus can improve overweight and non-alcoholic fatty liver disease in mice caused by high-fat and high-cholesterol diet by changing the intestinal flora, regulating the production of short-chain fatty acids, improving inflammatory response and lipid metabolism disorders [47]. Kaempferol is derived from C. pinnafitida. Naringenin chalcone is derived from Eubacterium ramulus. The GMrepo database revealed a significant enrichment of Eubacterium ramulus in obese individuals (LDA score = 3.042), suggesting its potential association with obesity progression. However, gutmgene database identified 17 bioactive SMs produced by this microorganism, including naringenin and phloretin, both of which have been validated to possess anti-obesity therapeutic potential [49,50].

Our toxicology prediction results using ProTox 3.0 showed that while quercetin may have mutagenic and carcinogenic potential, kaempferol appears to be non-toxic, and naringenin chalcone only demonstrated predicted immunotoxicity. However, these computational predictions contrast with established in vivo findings for quercetin. Multiple in vivo studies have consistently demonstrated that quercetin does not exhibit mutagenic activity, as evidenced by negative results in comprehensive genotoxicity assessments including micronucleus formation, chromosomal aberrations, sister chromatid exchange, unscheduled DNA synthesis, and alkali-labile DNA damage [51]. Furthermore, chronic toxicity studies in F344 rats revealed no significant carcinogenic effects even after continuous dietary administration of quercetin at concentrations up to 5.0% for 104 weeks [52]. Regarding naringenin chalcone, to date there are no published in vivo toxicity studies available, representing a significant knowledge gap in our understanding of this compound’s safety profile. This lack of experimental data highlights the need for future investigations to validate the immunotoxicity predicted by our computational analysis. While our study provides initial toxicity predictions using ProTox 3.0, these in silico results require further experimental verification through both in vitro and in vivo studies. Additionally, the potential organotoxicity risks associated with high-dose or prolonged administration (exceeding three years) of these SMs remain to be systematically investigated. Future research should therefore focus on comprehensive toxicological evaluations using graded concentrations and extended exposure periods to establish complete safety profiles.

In summary, our study clarified the potential mechanism and importance of Crataegus pinnatifida and gut microbiota as adjuvant therapy for obesity. However, we still need more basic experiments and clinical studies to confirm the effectiveness of Crataegus pinnatifida, gut microbiota and their SMs.

## 5. Limitations of the study

Our study used plant databases and microbial databases, and through bioinformatics methods, explored and organized Crataegus pinnatifida, obesity-related gut microbiota, SMs, targets, and pathways to explore their key roles in obesity treatment and provide a theoretical basis for clinical adjuvant treatment. However, as a data analysis study, our findings require further validation through in vitro and in vivo experiments to confirm the predicted mechanisms. In addition, this requires subsequent clinical treatment analysis of the gut microbiota we screened and Crataegus pinnatifida in appropriate proportions to obtain more accurate experimental results.

## 6. Conclusion

Our integrative approach using network pharmacology and bioinformatics predicts that targeting the PIK3R1 gene and modulating the PI3K/Akt signaling pathway can ameliorate obesity. The pathway can be targeted by SMs produced by Crataegus pinnatifida and by obesity-related gut microbiota. This prediction highlights the pivotal role of PIK3R1 in regulating adipogenesis, suggesting a promising avenue for obesity treatment. Our molecular docking and dynamics simulations identified quercetin, kaempferol and naringenin chalcone as potent binders to PIK3R1, opening new perspectives for microbiome-based therapies. These predictions require extensive in vitro and in vivo validation to translate these findings into clinical applications.

## Supporting information

S1 Table GO and KEGG analysis in network pharmacology. (XLSX)

S2 Table Differentially expressed genes. (XLSX)

S3 Table GO and KEGG analysis differentially expressed genes. (XLSX)

S4 Table Binding energy of molecular docking. (XLSX)

S1 File R language code used by the bioinformatics analysis. (DOCX)

## Abbreviations

SMs: secondary metabolites
MD: molecular docking
PIK3R1: Phosphoinositide-3-kinase regulatory subunit 1
PI3K: phosphoinositide 3-kinase
PKB: Protein Kinase B
GO: Gene Ontology
KEGG: Kyoto Encyclopedia of Genes and Genomes
DEGs: differentially expressed genes
GSEA: Gene Set Enrichment Analysis
LASSO: least absolute shrinkage and selection operator
ROC: receiver operating characteristic
ssGSEA: single-sample gene set enrichment analysis
QC: Quantum chemical
DCA: decision curve analysis
