# Supplementary material for: A blueprint of synergistic effect in Crataegus pinnatifida and obesity-related gut microbiota against obesity via systems biology concept
Source: PLoS One. 2025 Oct 1;20(10):e0332038. doi: 10.1371/journal.pone.0332038 (PMC12488023; doi:10.1371/journal.pone.0332038)
Supplement: S1 File — (DOCX) [file pone.0332038.s005.docx]

**Downloading and processing the dataset**

library(Biobase)

library(GEOquery)

GSE="GSE88837"

GPL="GPL570"

genename="Gene Symbol"

gset <- getGEO(GSE, GSEMatrix =T, getGPL = T, AnnotGPL = F)

if (length(gset) > 1) idx <- grep(GPL, attr(gset, "names")) else idx <- 1

gset <- gset[[idx]]

str(gset)

afexp<-data.frame(exprs(gset))

#annmatrix=rbind(ID=colnames(afexp),afexp)

#write.table(annmatrix,file="annmatrix.txt",sep="\t",quote=F,col.names = F)

head(gset@featureData@data)

nname=which(colnames(gset@featureData@data)==genename)

afexp$ID=as.character(gset@featureData@data[,nname])

ann=cbind(as.character(gset@featureData@data[,nname]),rownames(afexp))

write.table(ann,file="ann.xls",sep="\t",quote=F,col.names = F,row.names = F)

afexp<-afexp[afexp$ID!="",]

afexp=na.omit(afexp)

uniafexp<-aggregate(.~ID,afexp,mean)

write.table(uniafexp,file=paste0(GSE,".txt"),sep="\t",quote=F,col.names = T,row.names = F)

cli=pData(gset)

cliaf=rbind(ID=colnames(cli),cli)

write.table(cliaf,file="clinical.xls",sep="\t",quote=F,col.names = F)

**Differential analysis and WGCNA**

library(ggplot2)

library(limma)

library(pheatmap)

library(ggsci)

library(dplyr)

lapply(c('clusterProfiler','enrichplot','patchwork'), function(x) {library(x, character.only = T)})

library(org.Hs.eg.db)

library(patchwork)

library(WGCNA)

library(GSEABase)

library(randomcoloR)

library(AnnoProbe)

#if (!require('R.utils')) install.packages('R.utils')

R.utils::setOption( "clusterProfiler.download.method",'auto' )

library(GSVA)

GSE="GSE88837"

C="cont"

P="obesity"

Ccol= "#0073C2FF"

Pcol="#EFC000FF"

lowcol="#0073C2FF"

midcol="white"

highcol="#EFC000FF"

num=30

useDiff="T"

useDiffnum=5000

rt=read.table(paste0(GSE,".txt"),sep="\t",header=T,check.names=F)

rt=as.matrix(rt)

rownames(rt)=rt[,1]

exp=rt[,2:ncol(rt)]

dimnames=list(rownames(exp),colnames(exp))

rt=matrix(as.numeric(as.matrix(exp)),nrow=nrow(exp),dimnames=dimnames)

rt=avereps(rt)

rt[1:4,1:4]

a = rownames(rt)

b =data.frame(sapply(a,function(x) unlist(strsplit(x,'///'))[1]),

stringsAsFactors = F)

rownames(rt) = b[,1]

rt = avereps(rt)

sample=read.table("sample.txt",sep="\t",header=F,check.names=F,row.names = 1)

rt=rt[,rownames(sample)]

afcon=sum(sample[,1]==C)

max(rt)

if(max(rt)>50) rt=log2(rt+1)

rt1=normalizeBetweenArrays(as.matrix(rt))

cols=distinctColorPalette(ncol(rt))

pdf(file = "1.raw.pdf",width=15,height = 5)

par(cex = 0.7,mar=c(8,8,8,8))

if(ncol(rt)>40) par(cex = 0.5,mar=c(8,8,8,8))

boxplot(rt,las=2,col =cols )

dev.off()

cols=distinctColorPalette(ncol(rt))

pdf(file = "1.nor.pdf",width=15,height = 5)

par(cex = 0.5,mar=c(8,8,8,8))

if(ncol(rt1)>40) par(cex = 0.5,mar=c(8,8,8,8))

boxplot(rt1,las=2,col =cols )

dev.off()

rt2=rbind(ID=colnames(rt1),rt1)

write.table(rt2,file=paste0("1.","norexp_",GSE,".txt"),sep="\t",quote=F,col.names = F)

rt3=rbind(ID=colnames(rt),rt)

write.table(rt3,file=paste0("1.","rawexp_",GSE,".txt"),sep="\t",quote=F,col.names = F)

data=rt1

#data=rt

conData=data[,as.vector(colnames(data)[1:afcon])]

aftreat=afcon+1

treatData=data[,as.vector(colnames(data)[aftreat:ncol(data)])]

rt=cbind(conData,treatData)

conNum=ncol(conData)

treatNum=ncol(treatData)

save(rt,type,file = 'data.Rdata')

Type=c(rep("con",conNum),rep("treat",treatNum))

design <- model.matrix(~0+factor(Type))

colnames(design) <- c("con","treat")

fit <- lmFit(rt,design)

cont.matrix<-makeContrasts(treat-con,levels=design)

fit2 <- contrasts.fit(fit, cont.matrix)

fit2 <- eBayes(fit2)

Diff=topTable(fit2,adjust='fdr',number=length(rownames(data)))

max(Diff$logFC)

DIFFOUT=rbind(id=colnames(Diff),Diff)

write.table(DIFFOUT,file=paste0("2.","DIFF_all.xls"),sep="\t",quote=F,col.names=F)

diffSig=Diff[with(Diff, (abs(logFC)>0.585 & P.Value < 0.05 )), ]

diffSigOut=rbind(id=colnames(diffSig),diffSig)

write.table(diffSigOut,file=paste0("2.","DIFF_less.xls"),sep="\t",quote=F,col.names=F)

Diff=Diff[order(as.numeric(as.vector(abs(Diff$logFC))),decreasing = T),]

diffGene=as.vector(rownames(Diff))

diffLength=length(diffGene)

afGene=c()

if(diffLength>(2*num)){

afGene=diffGene[c(1:num,(diffLength-num+1):diffLength)]

}else{

afGene=diffGene

}

afExp=rt[afGene,]

afbiotype=data.frame(afGene,rep("1",length(afGene)))

colnames(afbiotype)=c("ID","Biotype")

biotype=annoGene(afGene, "SYMBOL")

rownames(biotype)=biotype[,1]

biotype=biotype[,2,drop=F]

afbiotype=afbiotype[c(which(afbiotype$ID %in% rownames(biotype)),c(1:length(afbiotype$Biotype))[-which(afbiotype$ID %in% rownames(biotype))] ),]

biotype=biotype[afbiotype$ID[1:length(biotype$biotypes)],,drop=F]

afbiotype$Biotype[1:length(biotype$biotypes)]=biotype$biotypes

afbiotype$Biotype[(length(biotype$biotypes)+1):length(afbiotype$Biotype)]=rep("NA",length(afbiotype$Biotype[(length(biotype$biotypes)+1):length(afbiotype$Biotype)]))

rownames(afbiotype)=afbiotype[,1]

afbiotype=afbiotype[,2,drop=F]

Type=c(rep(C,conNum),rep(P,treatNum))

names(Type)=colnames(rt)

Type=as.data.frame(Type)

anncolor=list(Type=c(C=Ccol,P=Pcol))

names(anncolor[[1]])=c(C,P)

pdf(file=paste0("3.", "DIFF_heatmap.pdf"),height=8,width=9)

pheatmap(afExp,

annotation_col=Type,

annotation_row=afbiotype,

color = colorRampPalette(c(lowcol,midcol,highcol))(50),

cluster_cols =F,

show_colnames = F,

scale="row",

fontsize = 10,

fontsize_row=6,

fontsize_col=8,

annotation_colors=anncolor

)

dev.off()

adjP=0.05

aflogFC=0.585

Significant=ifelse((Diff$P.Value<adjP & abs(Diff$logFC)>aflogFC), ifelse(Diff$logFC>aflogFC,"Up","Down"), "Not")

p = ggplot(Diff, aes(logFC, -log10(P.Value)))+

geom_point(aes(col=Significant),size=3)+

scale_color_manual(values=c(highcol, "#838B8B", lowcol))+

labs(title = " ")+

theme(plot.title = element_text(size = 16, hjust = 0.5, face = "bold"))+

geom_hline(aes(yintercept=-log10(adjP)), colour="gray", linetype="twodash",size=1)+

geom_vline(aes(xintercept=aflogFC), colour="gray", linetype="twodash",size=1)+

geom_vline(aes(xintercept=-aflogFC), colour="gray", linetype="twodash",size=1)

p

point.Pvalue=0.01

point.logFc=5

Diff$symbol=rownames(Diff)

pdf(paste0("3.", "DIFF_vol.pdf"),width=6.5,height=6)

p=p+theme_bw()

for_label <- Diff %>%

filter(abs(logFC) >point.logFc & P.Value< point.Pvalue )

p+geom_point(size = 1.5, shape = 1, data = for_label) +

ggrepel::geom_label_repel(

aes(label = symbol),

data = for_label,

color="black",

label.size =0.1

)

dev.off()

afdir <- paste0(getwd(),"/5.WGCNA")

dir.create(afdir)

traitData=sample

traitData[,2]=traitData[,1]

traitData[,1]=ifelse(traitData[,1]==C,1,0)

traitData[,2]=ifelse(traitData[,2]==P,1,0)

colnames(traitData)=c(C,P)

# The following setting is important, do not omit.

options(stringsAsFactors = FALSE)

#Read in the female liver data set

fpkm = read.table(paste0("1.rawexp_",GSE,".txt"),header=T,sep = "\t",check.names=F)

rownames(fpkm)=fpkm[,1]

if (sum(useDiff %in% c("TURE","T"))==1) {

fpkm=fpkm[intersect(rownames(Diff),rownames(fpkm))[1:useDiffnum],]

}

dim(fpkm)

names(fpkm)

datExpr0 = as.data.frame(t(fpkm[,-1]))

names(datExpr0) = fpkm[,1];

rownames(datExpr0) = names(fpkm[,-1])

datExpr0

gsg = goodSamplesGenes(datExpr0, verbose = 3)

gsg$allOK

if (!gsg$allOK)

{

# Optionally, print the gene and sample names that were removed:

if (sum(!gsg$goodGenes)>0)

printFlush(paste("Removing genes:", paste(names(datExpr0)[!gsg$goodGenes], collapse = ", ")))

if (sum(!gsg$goodSamples)>0)

printFlush(paste("Removing samples:", paste(rownames(datExpr0)[!gsg$goodSamples], collapse = ", ")))

# Remove the offending genes and samples from the data:

datExpr0 = datExpr0[gsg$goodSamples, gsg$goodGenes]

}

meanFPKM=0.5

n=nrow(datExpr0)

datExpr0[n+1,]=apply(datExpr0[c(1:nrow(datExpr0)),],2,mean)

datExpr0=datExpr0[1:n,datExpr0[n+1,] > meanFPKM] # for meanFpkm in row n+1 and it must be above what you set--select meanFpkm>opt$meanFpkm(by rp)

filtered_fpkm=t(datExpr0)

filtered_fpkm=data.frame(rownames(filtered_fpkm),filtered_fpkm)

names(filtered_fpkm)[1]="sample"

head(filtered_fpkm)

write.table(filtered_fpkm, file=paste0(afdir,"/FPKM_filter.xls"),row.names=F, col.names=T,quote=FALSE,sep="\t")

sampleTree = hclust(dist(datExpr0), method = "average")

pdf(file =paste0(afdir,"/1_sampleClustering.pdf"), width = 12, height = 9)

par(cex = 0.6)

par(mar = c(0,4,2,0))

plot(sampleTree, main = "Sample clustering to detect outliers", sub="", xlab="", cex.lab = 1.5,

cex.axis = 1.5, cex.main = 2)

### Plot a line to show the cut

##abline(h = 15, col = "red")

dev.off()

### Determine cluster under the line

##clust = cutreeStatic(sampleTree, cutHeight = 15, minSize = 10)

##table(clust)

### clust 1 contains the samples we want to keep.

##keepSamples = (clust==1)

##datExpr0 = datExpr0[keepSamples, ]

#Loading clinical trait data

for (df in colnames(traitData)) {

traitData[,df]=traitData[,df]/max(traitData[,df])

print(sd(traitData[,df]))

}

max(traitData)

dim(traitData)

names(traitData)

# remove columns that hold information we do not need.

allTraits = traitData

dim(allTraits)

names(allTraits)

# Form a data frame analogous to expression data that will hold the clinical traits.

fpkmSamples = rownames(datExpr0)

traitSamples =rownames(allTraits)

traitRows = match(fpkmSamples, traitSamples)

datTraits = allTraits[traitRows,]

rownames(datTraits)

collectGarbage()

# Re-cluster samples

sampleTree2 = hclust(dist(datExpr0), method = "average")

# Convert traits to a color representation: white means low, red means high, grey means missing entry

traitColors = numbers2colors(datTraits, signed = FALSE)

# Plot the sample dendrogram and the colors underneath.

#sizeGrWindow(12,12)

pdf(file=paste0(afdir,"/2_Sample dendrogram and trait heatmap.pdf"),width=12,height=11)

plotDendroAndColors(sampleTree2, traitColors,

groupLabels = names(datTraits),

main = "Sample dendrogram and trait heatmap",

marAll = c(1, 10, 3, 1))

dev.off()

#############################network constr########################################

# Allow multi-threading within WGCNA. At present this call is necessary.

# Any error here may be ignored but you may want to update WGCNA if you see one.

# Caution: skip this line if you run RStudio or other third-party R environments.

# See note above.

enableWGCNAThreads()

# Choose a set of soft-thresholding powers

powers = c(1:30)

# Call the network topology analysis function

sft = pickSoftThreshold(datExpr0, powerVector = powers, verbose = 5)

# Plot the results:

#sizeGrWindow(9, 5)

pdf(file=paste0(afdir,"/3_Scale independence.pdf"),width=9,height=5)

par(mfrow = c(1,2))

cex1 = 0.9

# Scale-free topology fit index as a function of the soft-thresholding power

plot(sft$fitIndices[,1], -sign(sft$fitIndices[,3])*sft$fitIndices[,2],

xlab="Soft Threshold (power)",ylab="Scale Free Topology Model Fit,signed R^2",type="n",

main = paste("Scale independence"));

text(sft$fitIndices[,1], -sign(sft$fitIndices[,3])*sft$fitIndices[,2],

labels=powers,cex=cex1,col="red");

# this line corresponds to using an R^2 cut-off of h

abline(h=0.9,col="red")

# Mean connectivity as a function of the soft-thresholding power

plot(sft$fitIndices[,1], sft$fitIndices[,5],

xlab="Soft Threshold (power)",ylab="Mean Connectivity", type="n",

main = paste("Mean connectivity"))

text(sft$fitIndices[,1], sft$fitIndices[,5], labels=powers, cex=cex1,col="red")

dev.off()

######chose the softPower

softPower =sft$powerEstimate

print(softPower)

adjacency = adjacency(datExpr0, power = softPower)

#======================================================ScaleFreeTopologyCheck And Histogram_k

# 1.

connectivity <- colSums(adjacency) - 1

# 2.

breaks <- seq(min(connectivity), max(connectivity), length.out = 30)

hist_k <- hist(connectivity, breaks = breaks, plot = FALSE)

k <- hist_k$mids

p_k <- hist_k$counts / sum(hist_k$counts)

# 3.

valid <- p_k > 0

k_valid <- k[valid]

p_k_valid <- p_k[valid]

# 4.

log_k_valid <- log10(k_valid)

log_p_k_valid <- log10(p_k_valid)

order_idx <- order(log_k_valid)

log_k_valid <- log_k_valid[order_idx]

log_p_k_valid <- log_p_k_valid[order_idx]

# 5.

fit <- lm(log_p_k_valid ~ log_k_valid)

r_squared <- summary(fit)$r.squared

slope <- coef(fit)[2]

subtitle <- paste("Scale R2 =", round(r_squared, 3), ", Slope =", round(slope, 3))

pdf(file=paste0(afdir,"/11_ScaleFreeTopologyCheck_Power.pdf"),width=5,height=5)

plot(log_k_valid, log_p_k_valid,

xlab = "log10(k)",

ylab = "log10(P(k))",

main = paste("Scale-free Topology Check\n",subtitle,sep=""),

pch = 20, col = "black")

abline(fit, col = "black", lwd = 2)

dev.off()

# 6.

pdf(file=paste0(afdir,"/12_Histogram_k_Power.pdf"),width=5,height=5)

hist(connectivity, breaks = breaks,

main = "Histogram of Connectivity (k)",

xlab = "Connectivity (k)",

ylab = "Frequency",

col = "grey", border = "black")

dev.off()

#======================================================

##### Turn adjacency into topological overlap

TOM = TOMsimilarity(adjacency);

dissTOM = 1-TOM

# Call the hierarchical clustering function

geneTree = hclust(as.dist(dissTOM), method = "average");

# Plot the resulting clustering tree (dendrogram)

#sizeGrWindow(12,9)

pdf(file=paste0(afdir,"/4_Gene clustering on TOM-based dissimilarity.pdf"),width=12,height=9)

plot(geneTree, xlab="", sub="", main = "Gene clustering on TOM-based dissimilarity",

labels = FALSE, hang = 0.04)

dev.off()

# We like large modules, so we set the minimum module size relatively high:

minModuleSize = 50

# Module identification using dynamic tree cut:

dynamicMods = cutreeDynamic(dendro = geneTree, distM = dissTOM,

deepSplit = 2, pamRespectsDendro = FALSE,

minClusterSize = minModuleSize);

table(dynamicMods)

# Convert numeric lables into colors

dynamicColors = labels2colors(dynamicMods)

table(dynamicColors)

# Plot the dendrogram and colors underneath

#sizeGrWindow(8,6)

pdf(file=paste0(afdir,"/5_Dynamic Tree Cut.pdf"),width=8,height=6)

plotDendroAndColors(geneTree, dynamicColors, "Dynamic Tree Cut",

dendroLabels = FALSE, hang = 0.03,

addGuide = TRUE, guideHang = 0.05,

main = "Gene dendrogram and module colors")

dev.off()

# Calculate eigengenes

MEList = moduleEigengenes(datExpr0, colors = dynamicColors)

MEs = MEList$eigengenes

# Calculate dissimilarity of module eigengenes

MEDiss = 1-cor(MEs);

# Cluster module eigengenes

METree = hclust(as.dist(MEDiss), method = "average")

# Plot the result

#sizeGrWindow(7, 6)

pdf(file=paste0(afdir,"/6_Clustering of module eigengenes.pdf"),width=7,height=6)

plot(METree, main = "Clustering of module eigengenes",

xlab = "", sub = "")

MEDissThres = 0.25

# Plot the cut line into the dendrogram

abline(h=MEDissThres, col = "red")

dev.off()

# Call an automatic merging function

merge = mergeCloseModules(datExpr0, dynamicColors, cutHeight = MEDissThres, verbose = 3)

# The merged module colors

mergedColors = merge$colors

# Eigengenes of the new merged modules:

mergedMEs = merge$newMEs

#sizeGrWindow(12, 9)

pdf(file=paste0(afdir,"/7_merged dynamic.pdf"), width = 9, height = 6.5)

plotDendroAndColors(geneTree, cbind(dynamicColors, mergedColors),

c("Dynamic Tree Cut", "Merged dynamic"),

dendroLabels = FALSE, hang = 0.03,

addGuide = TRUE, guideHang = 0.05)

dev.off()

# Rename to moduleColors

moduleColors = mergedColors

# Construct numerical labels corresponding to the colors

colorOrder = c("grey", standardColors(50))

moduleLabels = match(moduleColors, colorOrder)-1

MEs = mergedMEs

# Save module colors and labels for use in subsequent parts

#save(MEs, TOM, dissTOM, moduleLabels, moduleColors, geneTree, sft, file = "networkConstruction-stepByStep.RData")

##############################relate modules to external clinical triats######################################

# Define numbers of genes and samples

nGenes = ncol(datExpr0)

nSamples = nrow(datExpr0)

moduleTraitCor = cor(MEs, datTraits, use = "p")

moduleTraitPvalue = corPvalueStudent(moduleTraitCor, nSamples)

#sizeGrWindow(10,6)

pdf(file=paste0(afdir,"/8_Module-trait relationships.pdf"),width=7,height=7.5)

# Will display correlations and their p-values

textMatrix = paste(signif(moduleTraitCor, 2), "\n(",

signif(moduleTraitPvalue, 1), ")", sep = "")

dim(textMatrix) = dim(moduleTraitCor)

par(mar = c(10, 8.5, 3, 3))

# Display the correlation values within a heatmap plot

labeledHeatmap(Matrix = moduleTraitCor,

xLabels = names(datTraits),

yLabels = names(MEs),

ySymbols = names(MEs),

colorLabels = FALSE,

colors = blueWhiteRed(50),

textMatrix = textMatrix,

setStdMargins = FALSE,

cex.text = 0.5,

zlim = c(-1,1),

main = paste("Module-trait relationships"))

dev.off()

######## Define variable weight containing all column of datTraits

###MM and GS

# names (colors) of the modules

modNames = substring(names(MEs), 3)

geneModuleMembership = as.data.frame(cor(datExpr0, MEs, use = "p"))

MMPvalue = as.data.frame(corPvalueStudent(as.matrix(geneModuleMembership), nSamples))

names(geneModuleMembership) = paste("MM", modNames, sep="")

names(MMPvalue) = paste("p.MM", modNames, sep="")

#names of those trait

traitNames=names(datTraits)

geneTraitSignificance = as.data.frame(cor(datExpr0, datTraits, use = "p"))

GSPvalue = as.data.frame(corPvalueStudent(as.matrix(geneTraitSignificance), nSamples))

names(geneTraitSignificance) = paste("GS.", traitNames, sep="")

names(GSPvalue) = paste("p.GS.", traitNames, sep="")

####plot MM vs GS for each trait vs each module

##########example:royalblue and CK

#module="royalblue"

#column = match(module, modNames)

#moduleGenes = moduleColors==module

#trait="CK"

#traitColumn=match(trait,traitNames)

#sizeGrWindow(7, 7)

######

for (trait in traitNames){

traitColumn=match(trait,traitNames)

for (module in modNames){

column = match(module, modNames)

moduleGenes = moduleColors==module

if (nrow(geneModuleMembership[moduleGenes,]) > 1){

#sizeGrWindow(7, 7)

pdf(file=paste(afdir,"/9_", trait, "_", module,"_Module membership vs gene significance.pdf",sep=""),width=7,height=7)

par(mfrow = c(1,1))

verboseScatterplot(abs(geneModuleMembership[moduleGenes, column]),

abs(geneTraitSignificance[moduleGenes, traitColumn]),

xlab = paste("Module Membership in", module, "module"),

ylab = paste("Gene significance for ",trait),

main = paste("Module membership vs. gene significance\n"),

cex.main = 1.2, cex.lab = 1.2, cex.axis = 1.2, col = module)

dev.off()

}

}

}

#####

names(datExpr0)

probes = names(datExpr0)

#################export GS and MM###############

geneInfo0 = data.frame(probes= probes,

moduleColor = moduleColors)

for (Tra in 1:ncol(geneTraitSignificance))

{

oldNames = names(geneInfo0)

geneInfo0 = data.frame(geneInfo0, geneTraitSignificance[,Tra],

GSPvalue[, Tra])

names(geneInfo0) = c(oldNames,names(geneTraitSignificance)[Tra],

names(GSPvalue)[Tra])

}

for (mod in 1:ncol(geneModuleMembership))

{

oldNames = names(geneInfo0)

geneInfo0 = data.frame(geneInfo0, geneModuleMembership[,mod],

MMPvalue[, mod])

names(geneInfo0) = c(oldNames,names(geneModuleMembership)[mod],

names(MMPvalue)[mod])

}

geneOrder =order(geneInfo0$moduleColor)

geneInfo = geneInfo0[geneOrder, ]

write.table(geneInfo, file = paste0(afdir,"/10_GS_and_MM.xls"),sep="\t",row.names=F)

####################################################Visualizing the gene network#######################################################

nGenes = ncol(datExpr0)

nSamples = nrow(datExpr0)

# Transform dissTOM with a power to make moderately strong connections more visible in the heatmap

plotTOM = dissTOM^7

# Set diagonal to NA for a nicer plot

diag(plotTOM) = NA

nSelect = 1000

# For reproducibility, we set the random seed

set.seed(10)

select = sample(nGenes, size = nSelect)

selectTOM = dissTOM[select, select]

# There's no simple way of restricting a clustering tree to a subset of genes, so we must re-cluster.

selectTree = hclust(as.dist(selectTOM), method = "average")

selectColors = moduleColors[select]

# Open a graphical window

#sizeGrWindow(9,9)

# Taking the dissimilarity to a power, say 10, makes the plot more informative by effectively changing

# the color palette; setting the diagonal to NA also improves the clarity of the plot

plotDiss = selectTOM^7

diag(plotDiss) = NA

pdf(file=paste0(afdir,"/13_Network heatmap plot_selected genes.pdf"),width=9, height=9)

TOMplot(plotDiss, selectTree, selectColors, main = "Network heatmap plot, selected genes", col=gplots::colorpanel(250,'red',"orange",'lemonchiffon'))

dev.off()

####################################################Visualizing the gene network of eigengenes####################################################

#sizeGrWindow(5,7.5)

pdf(file=paste0(afdir,"/14_Eigengene dendrogram and Eigengene adjacency heatmap.pdf"), width=5, height=7.5)

par(cex = 0.9)

plotEigengeneNetworks(MEs, "", marDendro = c(0,4,1,2), marHeatmap = c(3,4,1,2), cex.lab = 0.8, xLabelsAngle= 90)

dev.off()

**GO and KEGG Enrichment Analysis**

library(GO.db)

library(org.Hs.eg.db)

library(clusterProfiler)

library(tidyverse)

library(ggplot2)

library(stringr)

library(enrichplot)

library(dplyr)

library(ggpubr)

sig_gene=read.table("gene.txt", header=T, sep="\t", check.names=F)

ids <- bitr(sig_gene$gene,'SYMBOL','ENTREZID','org.Hs.eg.db') #fromType = "SYMBOL",

sig_gene <- merge(sig_gene,ids,by.x='gene',by.y='SYMBOL')

gene_diff <- sig_gene$ENTREZID

kk=enrichGO(gene=gene_diff,OrgDb = "org.Hs.eg.db",pvalueCutoff = 1,qvalueCutoff = 1,ont="all",readable =T)

GO=as.data.frame(kk)

GO=GO[(GO$pvalue<0.05),]

write.table(GO,file = "GO_P0.05.txt",sep = "\t",quote = F,row.names = F)

save(GO,kk,file = "GO富集结果.Rdata")

load(file = "GO富集结果.Rdata")

pdf(file = "GO_P0.05_Top5气泡图.pdf",width=12,height = 8)

dotplot(kk, x = "GeneRatio", color = "pvalue", size = "Count",

showCategory =5,

label_format = 150,

orderBy="GeneRatio",

split = "ONTOLOGY") +

facet_grid(ONTOLOGY~., scale="free")+

scale_y_discrete(labels=function(x) str_wrap(x, width = 120))+

theme(

axis.text = element_text(size = 1, angle = 0, hjust = 1, vjust = 0.5),

)

dev.off()

kk1=enrichKEGG(gene=gene_diff,organism = "hsa",pvalueCutoff = 1,qvalueCutoff = 1)

library(DOSE)

kk1=setReadable(kk1,OrgDb = org.Hs.eg.db,keyType = "ENTREZID")

KEGG=as.data.frame(kk1)

KEGG=KEGG[(KEGG$pvalue<0.05),]

write.table(KEGG,file = "KEGG.txt-0.05",sep="\t",quote = F,row.names = F) save(KEGG,kk,file = "KEGG富集结果.Rdata")

load(file = "KEGG富集结果.Rdata")

pdf(file = "KEGG_P0.05_Top15气泡图.pdf",width=9,height = 6)

bub=dotplot(kk1,showCategory = 15,orderBy="GeneRatio",color = "pvalue",label_format = 100)

print(bub)

dev.off()

**Gene Set Enrichment Analysis**

library(presto)

library(stringr)

library(clusterProfiler)

library(msigdbr)

library(tidyverse)

library(enrichplot)

deg_data <- read.table("diff.txt", header = TRUE, sep = "\t", check.names = FALSE, row.names = 1)

deg_data$gene_symbol <- row.names(deg_data)

gene <- str_trim(deg_data$gene_symbol, "both")

gene <- bitr(gene, fromType = "SYMBOL", toType = "ENTREZID", OrgDb = "org.Hs.eg.db")

gene <- dplyr::distinct(gene, SYMBOL, .keep_all = TRUE)

gene_df <- data.frame(logFC = deg_data$logFC, SYMBOL = deg_data$gene_symbol)

gene_df <- merge(gene_df, gene, by = "SYMBOL")

geneList <- gene_df$logFC

names(geneList) <- gene_df$ENTREZID

geneList <- sort(geneList, decreasing = TRUE)

cat <- "C2"

subcat <- "KEGG"

m_df <- msigdbr(species = "Homo sapiens", category = cat, subcategory = subcat)

enrich_result <- GSEA(geneList, TERM2GENE = m_df[, c("gs_name", "entrez_gene")])

result <- enrich_result %>% as_tibble() %>% arrange(desc(NES))

write.csv(result, "GSEA_c2_kegg.csv")

saveRDS(enrich_result, "GSEA_c2_kegg.rds")

dotplot(enrich_result, color = "pvalue")

dotplot(enrich_result, split = ".sign") + facet_grid(~.sign)

dotplot(enrich_result, split = ".sign") + facet_wrap(~.sign, scales = "free")

library(enrichplot)

library(grid)

for (i in 1:nrow(result)) {

outFile <- paste0(result$ID[i], ".gseaplot.pdf")

p <- gseaplot2(enrich_result, result$ID[i], title = result$ID[i],

color = "#00468B", pvalue_table = FALSE, ES_geom = "line")

layout <- grid.layout(nrow = 1, ncol = 2, widths = unit.c(unit(0.7, "npc"), unit(0.3, "npc")))

grid.newpage()

pushViewport(viewport(layout = layout))

pushViewport(viewport(layout.pos.col = 1))

print(p)

pushViewport(viewport(layout.pos.col = 2))

grid.text(label = paste0("NES=", round(result$NES[i], 3), '\n',

"pvalue=", format(result$pvalue[i], scientific = TRUE, digits = 3), '\n',

"p.adjust=", format(result$p.adjust[i], scientific = TRUE, digits = 3)),

x = 0.55, y = 0.3, just = c("center", "center"), gp = gpar(col = '#ED0000', fontsize = 10))

recorded_plot <- recordPlot()

pdf(file = outFile, width = 8, height = 6)

replayPlot(recorded_plot)

dev.off()

}

**least absolute shrinkage and selection operator**

library(survival)

library(glmnet)

library(ggplot2)

library(ggsci)

library(patchwork)

library(limma)

inputFile="GSE88837.txt"

C="Healthy"

rt=read.table(inputFile, header=T, sep="\t", check.names=F)

rt=as.matrix(rt)

rownames(rt)=rt[,1]

exp=rt[,2:ncol(rt)]

dimnames=list(rownames(exp),colnames(exp))

data=matrix(as.numeric(as.matrix(exp)),nrow=nrow(exp),dimnames=dimnames)

data=avereps(data)

data=t(data)

a = read.table("disease.txt", header=F, sep="\t", check.names=F)[,1]

b = a[a%in%colnames(data)]

setdiff(a,b)

data=data[,b]

data=data[,b]

sample=read.table("sample.txt",sep="\t",header=F,check.names=F,row.names = 1)

data=data[rownames(sample),]

x=as.matrix(data)

afcon=sum(sample[,1]==C)

group=c(rep("0",afcon),rep("1",nrow(data)-afcon))

group=as.matrix(group)

rownames(group)=rownames(data)

y=as.matrix(group[,1])

set.seed(123)

cvfit = cv.glmnet(x, y,family = "binomial", nlambda=100, alpha=1,nfolds = 10) #这里alpha=1为

fit <- glmnet(x,y,family = "binomial")

cvfit$lambda.min

coef <- coef(fit, s = cvfit$lambda.min)

index <- which(coef != 0)

actCoef <- coef[index]

lassoGene=row.names(coef)[index]

geneCoef=cbind(Gene=lassoGene, Coef=actCoef)

write.table(geneCoef, file="geneCoef.xls", sep="\t", quote=F, row.names=F)

write.table(file="lassoset.txt",lassoGene,sep="\t",quote=F,col.names=F,row.names=F)

pdf("lasso.pdf",height = 5,width = 7)

layout(matrix(c(1,1,2,2), 2, 2, byrow = F)) #pdf("lambda.pdf")

plot(fit,xvar = 'lambda')

#dev.off()

#pdf("cvfit.pdf")

plot(cvfit)

abline(v=log(c(cvfit$lambda.min,cvfit$lambda.1se)),lty="dashed")

#dev.off()

dev.off()

**ROC and nomograms**

library(dplyr)

library(pROC)

library(ggplot2)

library(survival)

library(regplot)

library(rms)

library(ggsci)

library(survminer)

library(timeROC)

library(ggDCA)

library(limma)

inputFile="1.norexp_GSE88837.txt"

hub="gene.txt"

rt=read.table(inputFile, header=T, sep="\t", check.names=F)

rt=as.matrix(rt)

rownames(rt)=rt[,1]

exp=rt[,2:ncol(rt)]

dimnames=list(rownames(exp),colnames(exp))

data=matrix(as.numeric(as.matrix(exp)),nrow=nrow(exp),dimnames=dimnames)

data=avereps(data)

data=t(data)

sample=read.table("sample.txt",sep="\t",header=F,check.names=F)

colnames(sample)=c("ID","Type")

data=data[sample$ID,]

aSAH1=data[,read.table(hub, header=F, sep="\t", check.names=F)[,1]]

aSAH=cbind(sample,aSAH1)

aflist=roc(Type~G6PD+HSD11B1+RXRG+PIK3R1, data = aSAH)

g3 <- ggroc(aflist, size = 1.2,alpha=.6,)

g5=g3+ggsci::scale_color_lancet()

print(g5)

dd <- datadist(aSAH)

options(datadist="dd")

fit <- lrm(formula = Type ~ G6PD+HSD11B1+RXRG+PIK3R1, data =aSAH)

print(fit)

coef=as.data.frame(fit$coefficients)[-1,,drop=F]

coefout=cbind(ID=rownames(coef),coef)

write.table(coefout,file="coefficients.txt",sep="\t",quote=F,row.names = F)

pdf(file="nomogram.pdf", width=9, height=7.5)

plot(nomogram(fit,fun.at = seq(0.05,0.95,0.05)),funlabel = "nomogram model")

dev.off()

plot(regplot(fit,plots=c("density","boxes"), observation=T, title="Prediction Nomogram", clickable=T, points=TRUE,droplines=TRUE))

nomoscore=predict(fit, data=t(aSAH))

aSAH$nomoscore=nomoscore

write.table(aSAH,file="nomoscore.txt",sep="\t",quote=F,row.names = F)

library(limma)

rt=read.table("1.norexp_GSE88837.txt", header=T, sep="\t", check.names=F)

rt=as.matrix(rt)

rownames(rt)=rt[,1]

exp=rt[,2:ncol(rt)]

dimnames=list(rownames(exp), colnames(exp))

data=matrix(as.numeric(as.matrix(exp)), nrow=nrow(exp), dimnames=dimnames)

data=avereps(data)

data=data[rowMeans(data)>0,]

s1=read.table("s1.txt", header=F, sep="\t", check.names=F)

sampleName1=as.vector(s1[,1])

conData=data[,sampleName1]

s2=read.table("s2.txt", header=F, sep="\t", check.names=F)

sampleName2=as.vector(s2[,1])

treatData=data[,sampleName2]

data=cbind(conData, treatData)

conNum=ncol(conData)

treatNum=ncol(treatData)

Type=c(rep("cont",conNum),rep("obesity",treatNum))

colnames(data)=paste0(colnames(data),"_",Type)

gene=read.table("LASSCoregnostic genes", h.txt.txtr=F, sep="\t", check.names=F)

sameGene=intersect(as.vector(gene[,1]), rownames(data))

geneExp=data[sameGene,]

out=rbind(ID=colnames(geneExp),geneExp)

write.table(out,file="diagnosisExp.txt",sep="\t",quote=F,col.names=F)

library(rms)

library(rmda)

inputFile="diagnosisExp.txt"

data=read.table(inputFile, header=T, sep="\t", check.names=F, row.names=1)

data=t(data)

group=gsub("(.*)\\_(.*)", "\\2", row.names(data))

rt=cbind(as.data.frame(data), Type=group)

paste(colnames(data), collapse="+")

ddist=datadist(rt)

options(datadist="ddist")

lrmModel <- lrm(Type ~ RXRG + G6PD + PIK3R1 + HSD11B1, data = rt, x = TRUE, y = TRUE,maxit=1000)

nomo <- nomogram(lrmModel, fun = plogis, fun.at = c(0.000000000000000000000001,0.9), lp = FALSE, funlabel = "Risk of Disease")

cali=calibrate(lrmModel, method="boot", B=1000)

pdf("Calibration.pdf", width=6, height=6)

plot(cali,

xlab="Predicted probability",

ylab="Actual probability", sub=F)

dev.off()

rt$Type=ifelse(rt$Type=="cont", 0, 1)

dc=decision_curve(Type ~ RXRG + G6PD + PIK3R1 + HSD11B1, data=rt,

family = binomial(link ='logit'),

thresholds= seq(0,1,by = 0.01),

confidence.intervals = 0.95)

pdf(file="DCA.pdf", width=6, height=6)

plot_decision_curve(dc,

curve.names="Model",

xlab="Threshold probability",

cost.benefit.axis=T,

col="red",

confidence.intervals=FALSE,

standardize=FALSE)

dev.off()

pdf(file="clinical_impact.pdf", width=6, height=6)

plot_clinical_impact(dc,

confidence.intervals=T,

col = c("red", "blue"))

dev.off()

library(glmnet)

library(pROC)

expFile="diagnosisExp.txt"

geneFile="Core diagnostic genes.txt"

setwd("F:\\project\\xianyu\\02geneROC\\indepROC")

rt=read.table(expFile, header=T, sep="\t", check.names=F, row.names=1)

y=gsub("(.*)\\_(.*)", "\\2", colnames(rt))

y=ifelse(y=="cont", 0, 1)

geneRT=read.table(geneFile, header=F, sep="\t", check.names=F)

bioCol=rainbow(nrow(geneRT), s=0.9, v=0.9)

aucText=c()

k=0

for(x in as.vector(geneRT[,1])){

k=k+1

roc1=roc(y, as.numeric(rt[x,]))

if(k==1){

pdf(file="ROC.genes.pdf", width=6, height=6)

plot(roc1, print.auc=F, col=bioCol[k], legacy.axes=T, main="")

aucText=c(aucText, paste0(x,", AUC=",sprintf("%.3f",roc1$auc[1])))

}else{

plot(roc1, print.auc=F, col=bioCol[k], legacy.axes=T, main="", add=TRUE)

aucText=c(aucText, paste0(x,", AUC=",sprintf("%.3f",roc1$auc[1])))

}

}

legend("bottomright", aucText, lwd=2, bty="n", col=bioCol[1:(ncol(rt)-1)])

dev.off()

**Immune infiltration analysis**

library(reshape2)

library(ggpubr)

library(limma)

library(GSEABase)

library(GSVA)

expFile="1.norexp_GSE88837.txt"

load("TISIDB免疫浸润细胞基因集.rdata")

rt=read.table(expFile, header=T, sep="\t", check.names=F)

rt=as.matrix(rt)

rownames(rt)=rt[,1]

exp=rt[,2:ncol(rt)]

dimnames=list(rownames(exp),colnames(exp))

data=matrix(as.numeric(as.matrix(exp)),nrow=nrow(exp),dimnames=dimnames)

geneexp=avereps(data)

ssgseaScore=gsva(geneexp, tisidb_cell, method='ssgsea', kcdf='Gaussian', abs.ranking=TRUE)

normalize=function(x){

return((x-min(x))/(max(x)-min(x)))}

ssgseaScore=normalize(ssgseaScore)

ssgseaOut=rbind(id=colnames(ssgseaScore), ssgseaScore)

write.table(ssgseaOut,file="ssGSEA.cell.result.txt",sep="\t",quote=F,col.names=F)

rt=ssgseaOut[c(2:nrow(ssgseaOut)),]

rt=as.matrix(t(rt))

exp=rt[,1:ncol(rt)]

dimnames=list(rownames(exp),colnames(exp))

data=matrix(as.numeric(as.matrix(exp)),nrow=nrow(exp),dimnames=dimnames)

rt=as.data.frame(data)

group=read.table("sample.txt")

#rownames(group)=group[,1]

rt=rt[group[,1],]

rt$group=c(rep("cont",15),rep("obesity",15))

da=melt(rt, id.vars=c("group"))

colnames(da)=c("Group", "Immune", "Fraction")

da$Fraction=as.numeric(da$Fraction)

bioCol=c("#3D46F2","#F20505","#3A9B68","#EB8C30","#FF9900","#6E568C","#7CC767","#223D6C","#D20A13","#FFD121","#088247","#11AA4D")

bioCol=bioCol[1:length(levels(factor(da[,"Group"])))]

p=ggboxplot(da, x="Immune", y="Fraction", color="Group",

xlab="",

ylab="Immune infiltration",

legend.title="group",

palette=bioCol)

p=p+rotate_x_text(60)

pdf(file="ssgsea.cell.boxplot.pdf", width=10, height=6.5)

p+stat_compare_means(aes(group=Group),symnum.args=list(cutpoints = c(0, 0.001, 0.01, 0.05, 1), symbols = c("***", "**", "*", "")),label = "p.signif")

dev.off()

ssgsea=rt[,-29]

hmexp=t(ssgsea)

Group =c(rep("cont",15),rep("obesity",15))

annotation_col =as.data.frame(Group)

table(Group)

ann_colors = list(Group = c( cont = "#3298EB",obesity = "#C9414A"))

rownames(annotation_col) <- colnames(hmexp)

library(pheatmap)

pdf(file = "cell.heatmap.pdf", height = 6, width = 7)

pheatmap(hmexp,

annotation_col = annotation_col,

color =colorRampPalette(c("#1052CC","white","#B31616"))(60),

annotation_colors = ann_colors,

cluster_cols = F,

show_rownames = T,

show_colnames = F,

scale = "row",

fontsize = 10,

fontsize_row = 8,

fontsize_col = 3,

cellwidth =8,

cellheight = 11,

border = F

)

dev.off()

genelist=read.table("keycluster.txt")[,1]

immuscore <- function(gene){

y <- as.numeric(geneexp[gene,])

colnames <- colnames(ssgsea)

do.call(rbind,lapply(colnames, function(x){

dd <- cor.test(as.numeric(ssgsea[,x]),y,type="spearman")

data.frame(gene=gene,immune_cells=x,cor=dd$estimate,p.value=dd$p.value )

}))

}

immuscore("FOXP3")

data <- do.call(rbind,lapply(genelist,immuscore))

head(data)

write.table(data,file="cell.correlation.txt",sep="\t",quote=F,row.names=F)

data$pstar <- ifelse(data$p.value < 0.05,

ifelse(data$p.value < 0.01,"**","*"),

"")

data$pstar[1:20]

library(ggplot2)

library(dplyr)

ggplot(data, aes(gene,immune_cells)) +

geom_tile(aes(fill = cor), colour = "white",size=1)+

scale_fill_gradient2(low = "#2b8cbe",mid = "white",high = "#e41a1c")+

geom_text(aes(label=pstar),col ="black",size = 5)+

theme_minimal()+

theme(axis.title.x=element_blank(),

axis.ticks.x=element_blank(),

axis.title.y=element_blank(),

axis.text.x = element_text(angle = 45, hjust = 1),

axis.text.y = element_text(size = 8))+

labs(fill =paste0(" * p < 0.05","\n\n","** p < 0.01","\n\n","Correlation"))

ggsave("correlation.pdf", width = 5, height = 7)

dev.off()

**Chromosomal locations**

library(tidyverse)

library(circlize)

bed <- read_tsv("test.txt",col_names = T)

pdf(file="01.基因名称在里面.pdf",width=4,height=4)

circos.initializeWithIdeogram()

circos.genomicLabels(bed, labels.column = 4, side = "inside",

col = as.numeric(factor(bed[[1]])), line_col = as.numeric(factor(bed[[1]])))

dev.off()

pdf(file="02.基因名称在外面.pdf",width=6,height=6)

bed <- read_tsv("test.txt",col_names = T)

circos.initializeWithIdeogram(plotType = NULL)

circos.genomicLabels(bed,labels.column = 4,side = "outside",

connection_height=0.1,labels.side = side,

col = as.numeric(factor(bed[[1]])), line_col = as.numeric(factor(bed[[1]])))

set_track_gap(mm_h(1))

circos.trackPlotRegion(ylim = c(0,0.1),track.height = 0.05,bg.border="black",panel.fun = function(x, y) {

chr = CELL_META$sector.index

xlim = CELL_META$xlim

ylim = CELL_META$ylim

circos.text(mean(xlim),mean(ylim),chr,cex = 0.5,

col = "black",facing = "outside", niceFacing = TRUE)

})

circos.genomicIdeogram(track.height = mm_h(3))

dev.off()

**Box plot**

library(limma)

library(reshape2)

library(ggplot2)

library(ggpubr)

expFile="1.norexp_GSE88837.txt"

typeFile="sample.txt"

C="cont"

P="obesity"

Ccol="#0073C2FF"

Pcol= "#EFC000FF"

afmethod="wilcox.test"

rt=read.table(expFile, header=T, sep="\t", check.names=F)

rt=as.matrix(rt)

rownames(rt)=rt[,1]

exp=rt[,2:ncol(rt)]

table(rt>20)

dimnames=list(rownames(exp),colnames(exp))

data=matrix(as.numeric(as.matrix(exp)),nrow=nrow(exp),dimnames=dimnames)

data=avereps(data)

data=t(data)

keycluster=read.table("keycluster.txt", header=F, sep="\t", check.names=F)[,1]

data=data[,intersect(colnames(data),keycluster)]

type=read.table(typeFile, sep="\t", header=F, check.names=F, row.names=1)

colnames(type)="data"

sameSample=intersect(row.names(data),row.names(type))

rt1=cbind(data[sameSample,],type[sameSample,])

colnames(rt1)[ncol(rt1)]="data"

rt1=as.data.frame(rt1)

rt1[,1:(ncol(rt1)-1)]=lapply(rt1[,1:(ncol(rt1)-1)],as.numeric)

rt1=melt(rt1,id.vars=c("data"))

colnames(rt1)=c("data","Gene","Expression")

group=levels(factor(rt1$data))

rt1$data=factor(rt1$data, levels=c(C,P))

comp=combn(group,2)

my_comparisons=list()

for(j in 1:ncol(comp)){my_comparisons[[j]]<-comp[,j]}

boxplot=ggboxplot(rt1, x="Gene", y="Expression", fill ="data",

xlab="",

ylab="Experssion",

legend.title="Type",

width=0.8,

palette = c(Ccol,Pcol) )+

rotate_x_text(50)+

stat_compare_means(aes(group=data),

method=afmethod,

symnum.args=list(cutpoints=c(0, 0.001, 0.01, 0.05, 0.2,1), symbols=c("***", "**", "*", "#","ns")), label="p.signif")+

theme(axis.text=element_text(size=7,face = "bold"))

pdf(file="keycluster.diff.pdf", width=9, height=4)

print(boxplot)

dev.off()
